# Expression of glucose transporter 1 and prognosis in non-small cell lung cancer: a pooled analysis of 1665 patients

**DOI:** 10.18632/oncotarget.17604

**Published:** 2017-05-04

**Authors:** Zhibo Tan, Chao Yang, Xiaohan Zhang, Pingju Zheng, Weixi Shen

**Affiliations:** ^1^ Department of Oncology, Shenzhen Hospital of Southern Medical University, Shenzhen 518110, Guangdong, PR China; ^2^ Department of Gastroenterology, Shenzhen Hospital of Southern Medical University, Shenzhen 518110, Guangdong, PR China

**Keywords:** GLUT1, meta-analysis, overall survival, prognosis

## Abstract

Glucose transporter 1 (GLUT1) plays an important role in the transport and metabolism of glucose in cancer cells. An increasing number of studies have explored the connection between GLUT1 expression and prognosis in non-small cell lung cancer (NSCLC), but the results have been controversial. Therefore, we conducted a meta-analysis to obtain a comprehensive evaluation of the prognostic value of GLUT1 in NSCLC. Relevant studies from PubMed, Embase, and Web of Science were searched. Hazard ratios (HRs) and odds ratios (ORs) with their 95% confidence intervals (CIs) were used as the effective measures. A total of 10 studies involving 1,665 patients were included in this meta-analysis. The results showed that GLUT1 overexpression was associated with poor overall survival (HR = 2.21; 95% CI, 1.42–3.42; p < 0.001) and disease-free survival (HR = 1.73; 95% CI, 1.35–2.23; p < 0.001). Furthermore, elevated GLUT1 expression correlated with sex (OR = 2.29; 95% CI, 1.17–4.49; p = 0.015), advanced tumor stage (OR = 2.46; 95% CI, 1.79–3.38; p < 0.001), histology (OR = 6.99; 95% CI, 4.71–10.38; p < 0.001), and large tumor size (OR = 2.77; 95% CI, 1.73–4.44; p < 0.001). This meta-analysis revealed overexpression of GLUT1 to be a biomarker of worse prognosis in NSCLC.

## INTRODUCTION

Lung cancer is a lethal cancer with the highest incidence among all cancer types worldwide [1]. Non-small cell lung cancer (NSCLC) accounts for approximately 85% of all lung cancer cases [2]. In recent years, great progresses have been made in terms of treatment strategies for NSCLC [3, 4]; however, long-term survival remains unsatisfactory with a 5-year survival rate as low as 16.3%[2, 5]. The fact that prognostic parameters are lacking is one important reason for the disappointing prognosis [5]. Several clinical features, including pathological stage, gene mutational status [4], and smoking history have been used as biomarkers for prognostication. However, they only provide crude measures of the aggressiveness of NSCLC. Therefore, novel biological markers are still needed.

Glucose is the major source of energy for cells [6]. Compared to normal cells, cancer cells often have higher rates of glucose metabolism to support their rapid proliferation [7]. The glucose transporter (GLUT) family is responsible for glucose uptake [8]. The GLUT family is a collection of 14 membrane proteins [8], among which glucose transporter 1 (GLUT1, also known as SLC2A1) was the first to be cloned and has been the most extensively studied [9]. GLUT1 overexpression has been reported in a large variety of malignancies, including prostate cancer, thyroid cancer, gastric cancer, head and neck cancer, and NSCLC [6]. Previous studies [10-12] have presented controversial and even contrary results regarding the prognostic role of GLUT1 in NSCLC. For example, Minami et al. [10] showed that GLUT1 expression was a prognostic factor for poor survival; however, Osugi et al. [13] reported no significant correlation between GLUT1 and overall survival (OS) in NSCLC. These inconsistent results may be caused by the limited sample sizes in each single study. Therefore, we conducted a meta-analysis to clarify the prognostic significance of GLUT1 expression in NSCLC.

## RESULTS

### Study selection process and characteristics of included studies

**Figure 1:**
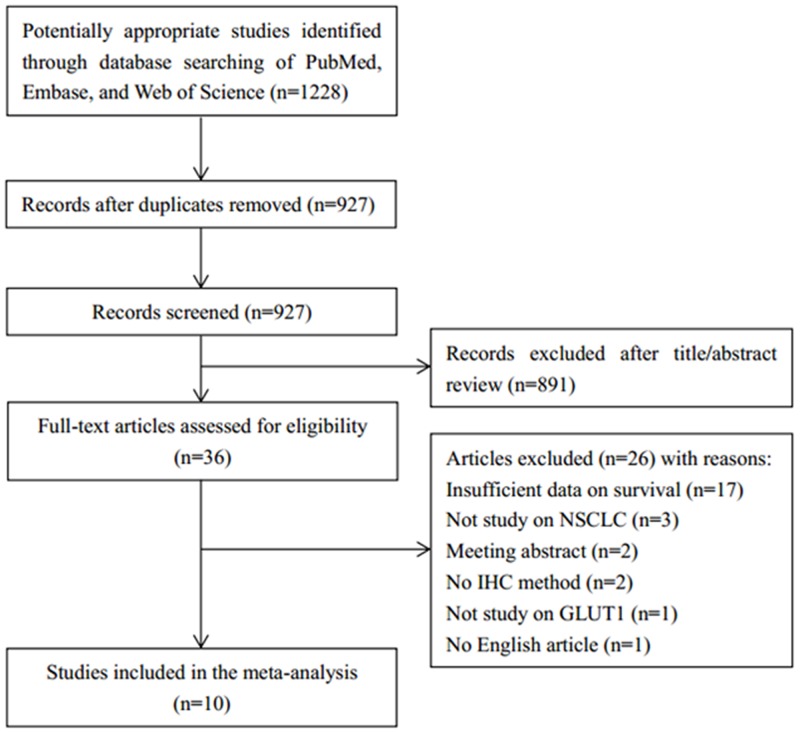
Flow chart demonstrating those studies that were processed for inclusion in the meta-analysis

The selection process of eligible studies is shown in Figure 1. Initial electronic search identified 1228 records, and after duplicates were removed, 927 remained for selection. A total of 891 records were excluded by title/abstract screening. Subsequently, 36 studies were evaluated by full-text reading. Twenty-six studies were further excluded because of inadequate data, absence of immunohistochemistry (IHC) method, being a meeting abstract, or not published in English. Finally, a total of 10 studies [10-19] involving 1,665 patients were included in the meta-analysis. The baseline characteristics of the included studies are listed in Table 1. Six studies [10, 12, 13, 15, 17, 18] were from Japan, and one each from Norway [11] The Netherlands [14] Greece [16], and Korea [19]. All included studies employed retrospective study designs, and were of high quality with Newcastle-Ottawa Scale (NOS) scores of 7 to 9.

**Table 1:** Characteristics of the included studies.

Study	Year	Country	Ethnicity	Age(years) median(range)	Histological type	Sample size	Gender (M/F)	Study design	Tumor stage	Study period	Method	NOS score	Outcome measured
Minami	2002	Japan	Asian	64(41-81)	Adenocarcinoma	47	28/19	Retrospective	I	1991-1997	IHC	9	OS
Andersen	2011	Norway	Caucasian	67(28-85)	Mixed	335	253/82	Retrospective	I -III	1990-2004	IHC	8	DFS
Kaira	2011	Japan	Asian	67(39-89)	Mixed	160	97/63	Retrospective	I -III	2002-2004	IHC	7	OS
Meijer	2012	The Netherlands	Caucasian	NR	Mixed	84	51/33	Retrospective	I -III	2002-2008	IHC	8	DFS
Sasaki	2012	Japan	Asian	65(29-86)	Mixed	283	189/94	Retrospective	I -IV	2001-2008	IHC	8	OS
Karpathiou	2013	Greece	Caucasian	67(28-83)	Mixed	115	103/12	Retrospective	I -IV	NR	IHC	8	OS
Maki	2013	Japan	Asian	65(29-83)	Adenocarcinoma	105	49/56	Retrospective	I	2004-2006	IHC	7	OS/DFS
Furukawa	2015	Japan	Asian	65	Mixed	133	90/43	Retrospective	I -III	2007-2010	IHC	8	OS/DFS
Osugi	2015	Japan	Asian	70(48-87)	Mixed	134	92/42	Retrospective	I -III	1998-2000	IHC	8	OS/DFS
Koh	2017	Korea	Asian	64(35-86)	Mixed	269	189/80	Retrospective	I -III	2009-2013	IHC	8	OS/DFS

Abbreviations: IHC, immunohistochemistry; NR, not reported; OS, overall survival; DFS, disease-free survival; NOS, Newcastle-Ottawa Scale.

### Correlation of GLUT-1 with overall survival

**Figure 2:**
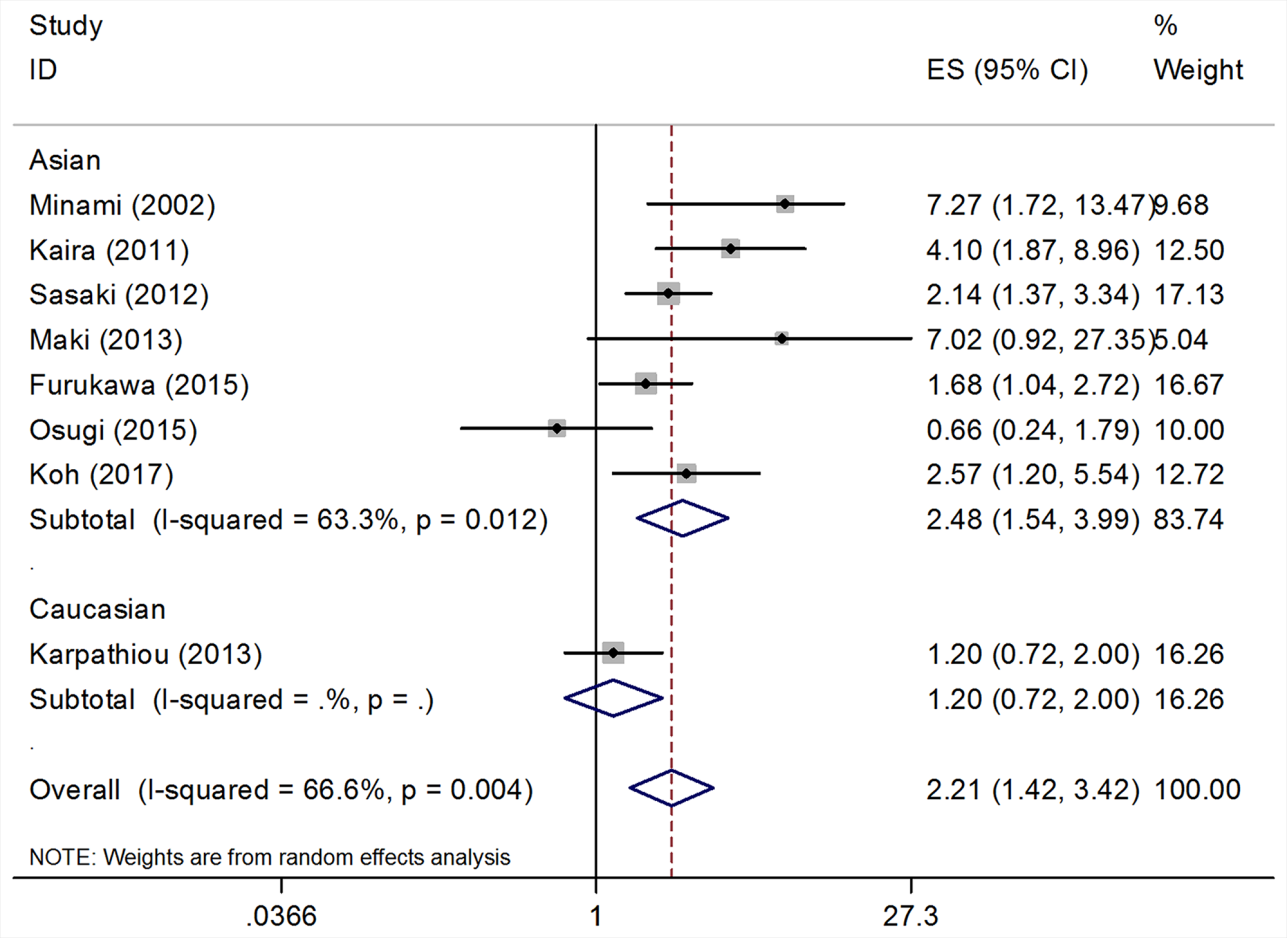
Forest plot of the association between GLUT1 and OS, subgroup analysis was stratified by ethnicity

A total of eight studies containing 1,246 patients explored the relationship between GLUT1 expression and OS. The pooled HR was 2.21 with a 95% CI of 1.42–3.42 (p < 0.001; Table 2, Figure 2), although significant heterogeneity (*I*^2^ = 66.6%; P_h_ = 0.004) was detected. Subgroup analysis stratified by ethnicity showed that high GLUT1 expression remained a significant indicator for poor OS in Asian patients (HR = 2.48; 95% CI, 1.54–3.99; p < 0.001; Table 2, Figure 2); however, no significant association was found for Caucasian patients (HR = 1.2; 95% CI, 0.72–2.00; p = 0.485; Table 2, Figure 2).

**Table 2:** Meta-analysis: HR values of OS and DFS in NSCLC subgroups according to patient source.

Factors	No. of studies	No. of patients	Effects model	HR (95%CI)	p	Heterogeneity	
						*I*^2^(%)	P_h_
Overall for OS	8	1246	REM	2.21(1.42-3.42)	<0.001	66.6	0.004
Ethnicity							
Asian	7	1131	REM	2.48(1.54-3.99)	<0.001	63.3	0.012
Caucasian	1	115	-	1.2(0.72-2)	0.485	-	-
Overall for DFS	6	1060	FEM	1.73(1.35-2.23)	<0.001	0	0.58
Ethnicity							
Asian	4	641	FEM	1.61(1.2-2.16)	0.001	0	0.479
Caucasian	2	419	FEM	2.16(1.3-3.57)	0.003	0	0.558

### Association between GLUT1 and disease-free survival

**Figure 3:**
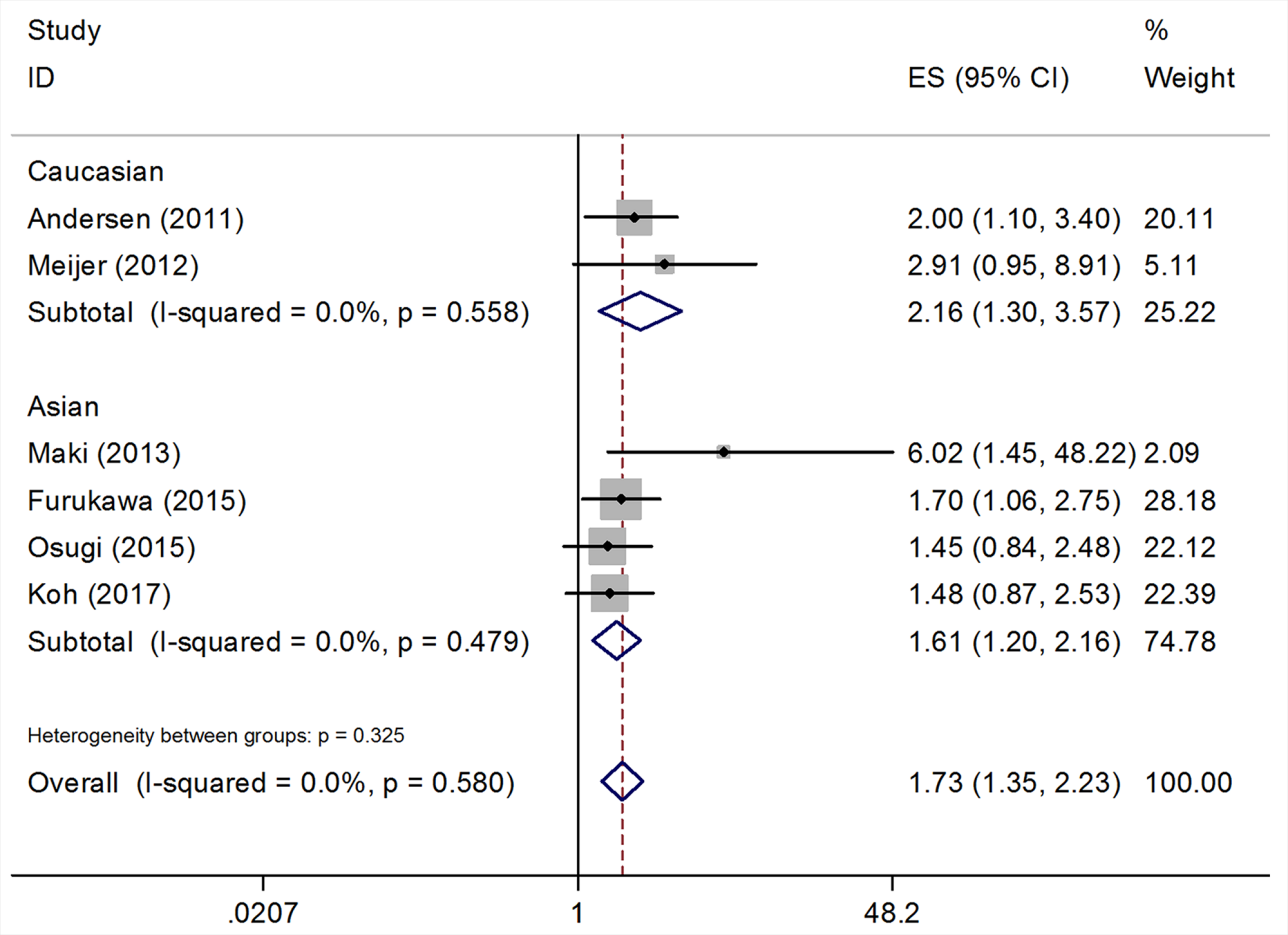
Forest plot of the association between GLUT1 and DFS, subgroup analysis was stratified by ethnicity

Pooled data from six studies with 1,060 patients with no heterogeneity (*I*^2^ = 0; P_h_ = 0.58) indicated that GLUT1 expression was predictive for poor disease-free survival (DFS) (HR = 1.73; 95% CI, 1.35–2.23; p < 0.001; Table 2, Figure 3). Subgroup analysis demonstrated that GLUT1 was still a biomarker for poor DFS in both Asian (HR = 1.61; 95% CI, 1.2–2.16; p = 0.001) and Caucasian (HR = 2.16; 95% CI, 1.3–3.57; p = 0.003) patients.

### Correlation of GLUT-1 with clinicopathological parameters

The association between GLUT1 and six clinicopathological parameters were investigated. The clinicopathological parameters were sex (male vs. female), tumor stage (III - IV vs. I - II), age (≥65 years vs. <65 years), histology (squamous cell carcinoma, SCC vs. adenocarcinoma), tumor size (≥3 cm vs. <3 cm), and differentiation (poor vs. moderate/well). The results of pooled analyses are summarized in Table 3. The combined data showed that GLUT1 expression significantly correlated with male sex (OR = 2.29; 95% CI, 1.17–4.49; p = 0.015), advanced tumor stage (OR = 2.46; 95% CI, 1.79–3.38; p < 0.001), histology (OR = 6.99; 95% CI, 4.71–10.38; p < 0.001), and large tumor size (OR = 2.77; 95% CI, 1.73–4.44; p < 0.001) (Table 3). However, there was no significant connection between GLUT1 expression and age (OR = 1.24; 95% CI, 0.9–1.72; p = 0.19) or differentiation (OR = 1.03; 95% CI, 0.15–6.96; p = 0.976).

**Table 3:** Association between GLUT1 and clinicalpathological characteristics.

Variables	No. of studies	No. of patients	Effects model	OR (95%CI)	p	Heterogeneity		Publication bias Begg’s p
						*I*^2^(%)	P_h_	
Gender (male vs. female)	5	721	REM	2.29(1.17-4.49)	0.015	69.5	0.011	1
Tumor stage (III-IV vs. I - II)	5	885	FEM	2.46(1.79-3.38)	<0.001	49.7	0.093	0.221
Age (years, ≥65 vs. <65)	4	606	FEM	1.24(0.9-1.72)	0.19	8.9	0.349	0.734
Histology (SCC vs. adenocarcinoma)	4	616	FEM	6.99(4.71-10.38)	<0.001	50	0.112	0.308
Tumor size (≥3cm vs. <3cm)	3	323	FEM	2.77(1.73-4.44)	<0.001	0	0.48	1
Differentiation (poor vs. moderate/well)	3	333	REM	1.03(0.15-6.96)	0.976	91.8	<0.001	0.602

Abbreviations: REM, random-effects model; FEM, fixed-effects model; SCC, squamous cell carcinoma.

### Publication bias

**Figure 4:**
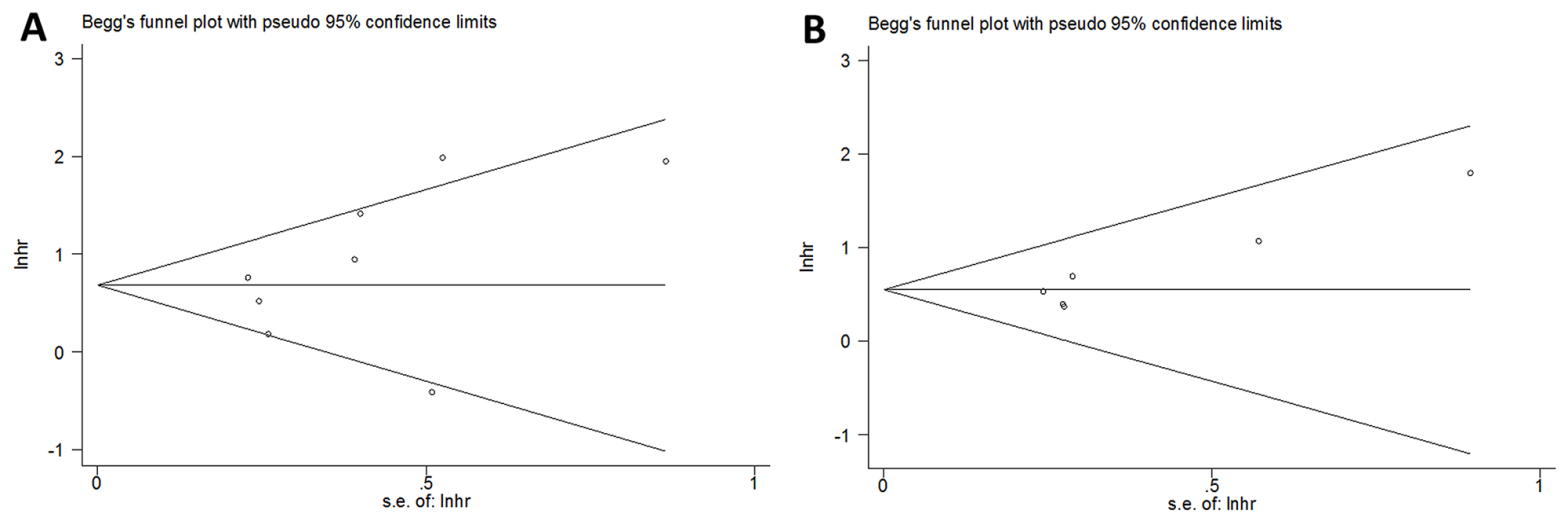
Publication bias tested by Begg’s funnel plot for **(A)** OS, p=0.386 and **(B)** DFS, p=0.133

Begg’s funnel plot was carried out to assess publication bias. The p-values of Begg’s test for analysis of OS and DFS were 0.386 and 0.133, respectively. Moreover, the funnel plots (Figure 4) were visually symmetrical. The Begg’s p-values for analysis of clinicopathological parameters were greater than 0.05 (Table 3). Therefore, there was no evidence of significant publication bias in this meta-analysis.

## DISCUSSION

A number of studies have explored the prognostic significance of GLUT1 in NSCLC; however, results have been inconsistent, which prompted this meta-analysis to obtain an objective view of this issue. The present meta-analysis extracted data from 10 eligible studies for the association of GLUT1 expression with OS, DFS, and clinicopathological characteristics. The results demonstrated that GLUT1 was an indicator for worse OS and DFS. In addition, GLUT1 expression significantly correlated with sex, tumor stage, histology, and tumor size in patients with NSCLC. These results suggest that GLUT1 could act as a risk factor involved in aggressive biological behaviors of tumor cells, and a predictor of shortened survival of NSCLC patients.

GLUT1 is a key rate-limiting factor in the transport and metabolism of glucose in cancer cells [20]. Cancer cells are highly proliferative due to disease progression. In various cancers, hypoxia often occurs when tumor cells outstrip their vasculature [6]. Therefore, genes regulating glycolysis and glucose transport are activated to provide energy [21]. GLUT1 overexpression was found in a series of solid tumors,[6] and the prognostic value of GLUT1 for survival has also been reported. Jans et al. [22] showed that the presence of cytoplasmic GLUT-1 was a prognosticator for shorter recurrence-free survival in prostate cancer patients. Kunkel et al. [23] indicated that GLUT1 was a significant negative biomarker of OS in patients with oral squamous cell carcinoma. Moreover, Amann et al. [20] found that GLUT1 expression was increased in hepatocellular carcinoma, and was associated with advanced tumor stages. In the current meta-analysis, we found that high GLUT1 expression was connected with poor OS and DFS in NSCLC, which was consistent with previous findings in other types of cancer [9, 20, 22, 23]. To our knowledge, this is the first meta-analysis of the prognostic value of GLUT1 in NSCLC. Furthermore, we also demonstrated that GLUT1 was associated with advanced tumor stage and large tumor size. This association may be due to advanced stage and large tumors have high rates of glucose metabolism needed for energy production and therefore necessitating extensive overexpression and activation of GLUT1.

To guarantee study homogeneity, we only included studies using IHC to detect GLUT1 expression. We also noted that one study reported GLUT1 gene amplification to be significantly correlated with shorter survival in lung cancer patients (p < 0.01) [24], and remained a statistically significant prognostic factor in a multivariate analysis. These findings are consistent with our results that demonstrate histological expression of GLUT1 as a prognostic biomarker of survival. Taken together, the results suggest that high GLUT1 expression at both transcript and protein levels have prognostic value.

Several limitations must be taken into account in this meta-analysis. First, significant heterogeneity was observed for the analysis of GLUT1 and OS. Specifically, substantial heterogeneity was found in the OS analysis in general and in the subgroup of Asian ethnicity. Although we selected eligible studies using uniform criteria, heterogeneity still existed in the primary studies. Second, the included studies were classified as Asian and Caucasian groups regarding ethnicity; however, in the Asian group, all studies were from one Asian country, Japan. Therefore, the Asian subgroup could be overrepresented, because other Asian countries such as China and Korea were not included. Studies from other Asian countries are needed for a comprehensive evaluation of GLUT1 expression in NSCLC. Third, the relationship between GLUT1 expression and survival according to histological types was not analyzed in this meta-analysis. However, of the analyzed studies, two [10, 17] included only adenocarcinoma, whereas the remaining [11-16, 18, 19] included patients with different histological types. The survival outcomes of different histologic types could not be extracted because of insufficient data.

In summary, this meta-analysis showed that GLUT1 overexpression was a biomarker of worse OS and DFS in NSCLC. In addition, elevated GLUT1 expression significantly correlated with advanced tumor stage and large tumor size. We conclude that GLUT1 may serve as a prognostic biomarker for NSCLC. However, because of some limitations, further large prospective studies are needed to validate our results.

## METHODS

### Search strategy

This study was performed in accordance with Preferred Reporting Items for Systematic Reviews and Meta-Analyses (PRISMA) guidelines [25]. The electronic databases of PubMed, Embase, and Web of Science were searched till February 2017. The following search terms were used; “glucose transporter-1”, “GLUT-1”, “SLC2A1”, “non-small cell lung cancer”, “NSCLC”, “lung carcinoma”, and “lung neoplasms”. Furthermore, reference lists of relevant studies were checked for potential eligible studies.

### Inclusion and exclusion criteria

Eligible studies must meet the following inclusion criteria: (1) the diagnosis of NSCLC was histopathologically proven; (2) GLUT1 expression was detected by IHC method; (3) the correlation between GLUT1 expression and OS or disease-free survival (DFS) was evaluated or sufficient data was provided for calculation; and (4) studies as full-text articles were published in English. The exclusion criteria were as follows: (1) reviews, meeting abstract, letters, or duplicate publications; (2) animal studies; and (3) studies with insufficient data.

### Data extraction and quality assessment

Two investigators (ZBT and WXS) independently extracted the following information from eligible studies: first author’s surname, publication year, study location, age, sample size, tumor stage, histological type, study design, detection method, survival outcomes measured, and study period. The qualities of included studies were evaluated according to the Newcastle-Ottawa Scale (NOS). A score of 0 - 9 was used to indicate the quality of each study, on basis of selection (0 - 4), comparability (0 - 2), and outcome (0 - 3). Studies labeled with ≥7 were considered as high quality studies.

### Statistical analysis

To evaluate the impact of GLUT1 expression on OS and DFS, hazard ratio (HR) with its relative 95% confidence interval (CI) was used in combination as the effective measure. A pooled HR >1 indicated a worse OS/DFS in high GLUT1 expressing patients, and a pooled HR <1 represented the opposite results. Odds ratios (ORs) and their 95% CIs were used to assess the correlation between GLUT1 overexpression and clinicopathological variables, including sex, age, tumor stage, histology, tumor size, and differentiation. Heterogeneity across studies was evaluated using Cochran’s Q test and *I*^2^ statistics. An *I*^2^ > 50% and p-value for heterogeneity <0.10 indicated significant heterogeneity, and a random-effects was used for calculations, otherwise, a fixed-effects model was adopted. Publication bias was evaluated using Begg’s funnel plot. A p-value <0.05 was considered statistically significant. All statistical analyses were performed using Stata 12.0 software (Stata Corporation, College Station, TX, USA).

## Abbreviations

GLUT1: Glucose transporter 1
NSCLC: Non-small cell lung cancer
HR: Hazard ratio
OR: Odds ratio
CI: Confidence interval
OS: Overall survival
NOS: Newcastle-Ottawa Scale
IHC: Immunohistochemistry
PRISMA: Preferred Reporting Items for Systematic Reviews and Meta-Analyses
